# EUS-guided hepaticogastrostomy using a rendezvous technique to treat left intrahepatic duct stones in a patient with recurrent pyogenic cholangitis

**DOI:** 10.1016/j.vgie.2021.11.006

**Published:** 2022-01-05

**Authors:** Michael Lajin

**Affiliations:** SHARP Health, San Diego, California

## Abstract

Video 1 EUS-guided hepaticogastrostomy using a rendezvous technique to enable cholangioscopy and electrohydraulic lithotripsy of left intrahepatic duct stones.

EUS-guided hepaticogastrostomy using a rendezvous technique to enable cholangioscopy and electrohydraulic lithotripsy of left intrahepatic duct stones.

## Introduction

The rendezvous technique to facilitate difficult biliary cannulation has been described.^1^ A temporary EUS-guided hepaticogastrostomy followed by staged antegrade cholangioscopy and electrohydraulic lithotripsy has been described for the management of recurrent pyogenic cholangitis in patients with altered foregut anatomy.^2^ However, penetrating the gastric wall to create the hepaticogastrostomy tract can prove challenging without the assistance of electrosurgical current.^3^

We describe the use of a salvage rendezvous technique to facilitate hepaticogastrostomy tract creation for the treatment of recurrent pyogenic cholangitis in a patient with native foregut anatomy when the conventional technique was initially unsuccessful.

## Case description

**Figure 1:**
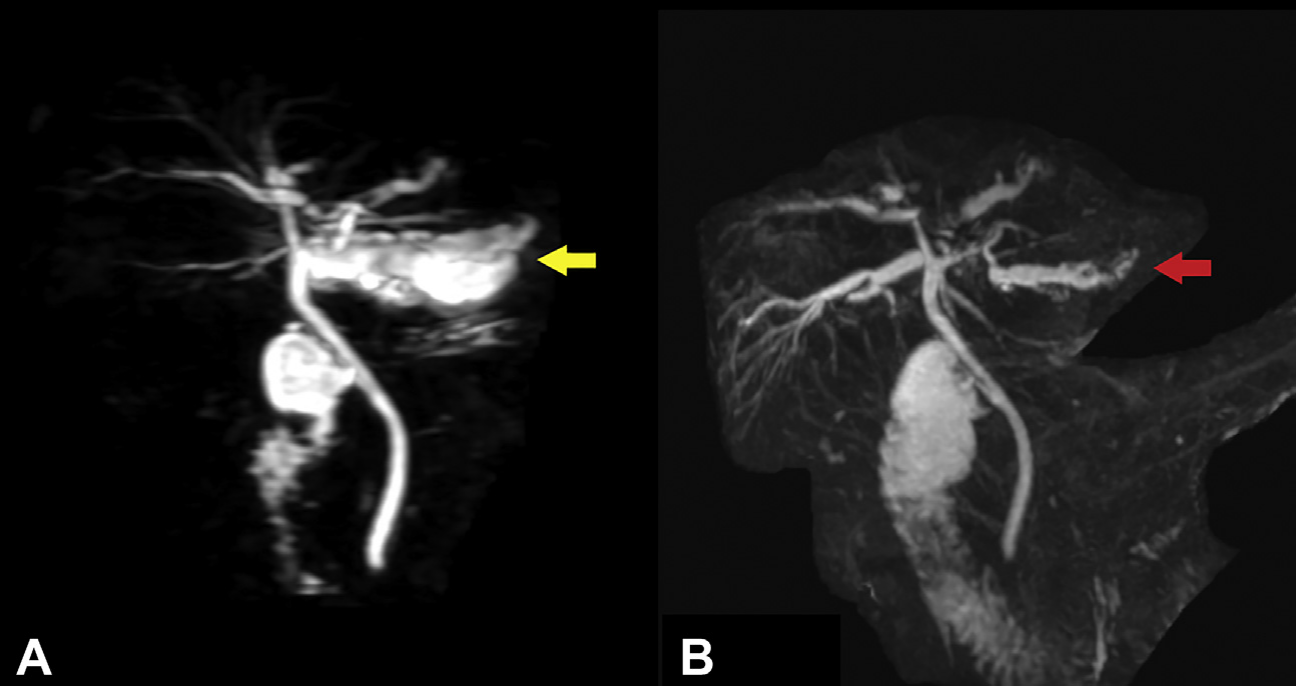
**A,** Preprocedural MRCP image showing intrahepatic ductal dilation of the lateral segment of the left liver lobe with stones (*yellow arrow*). **B,** Postprocedural MRCP image showing decompression of the duct and clearance from stones (*red arrow*).

**Figure 2:**
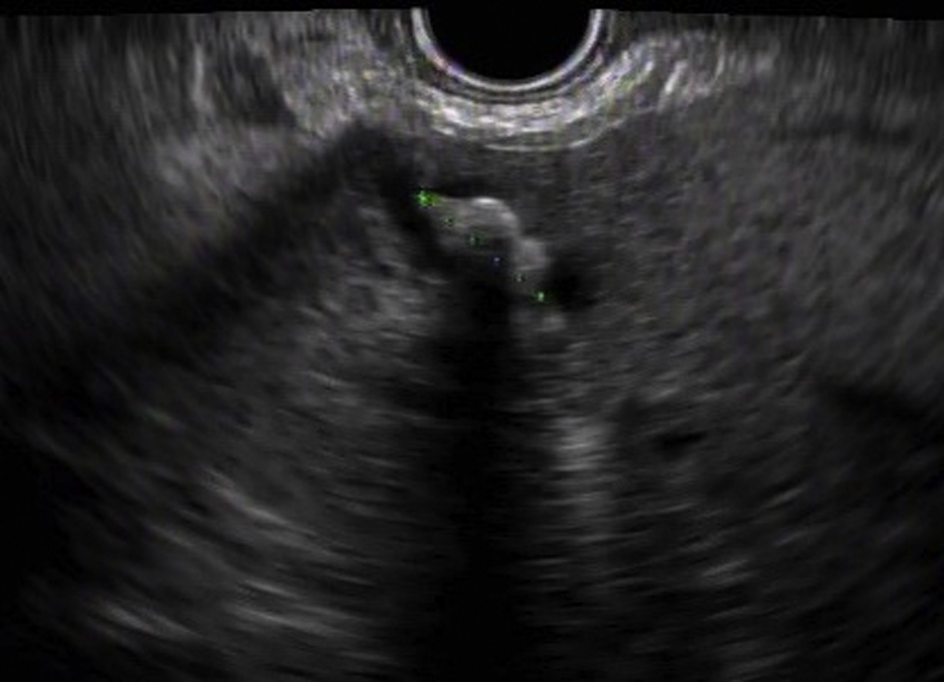
EUS image showing an intrahepatic ductal stone.

A 51-year-old man presented with abdominal pain and leukocytosis. MRCP showed severe intrahepatic ductal dilatation involving the lateral segment of the left hepatic lobe, with multiple intraductal stones (Fig. 1A). EUS showed large stones located in the dilated peripheral ducts of segment III (Fig. 2). A transpapillary cholangioscope was unable to reach the target peripheral ducts because of a sharp angulation and resistance encountered.

**Figure 3:**
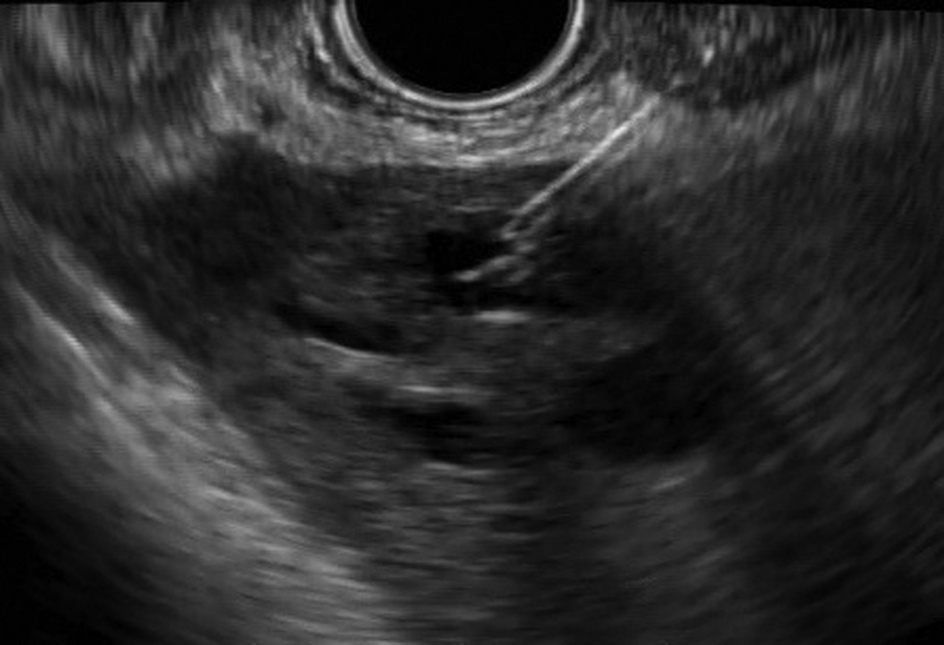
Puncturing the targeted duct of segment III with a needle.

A linear echoendoscope was advanced to the stomach facing the lesser curvature. The peripheral duct at segment III was punctured with a 19-gauge needle (Fig. 3). A cholangiogram was obtained, and a wire then was coiled inside the left intrahepatic ducts. Many attempts to dilate the hepaticogastrostomy tract using a balloon catheter and ERCP catheter failed because of the inability of these devices to penetrate the gastric wall (a 6F coaxial electrocautery dilator was not available).

**Figure 4:**
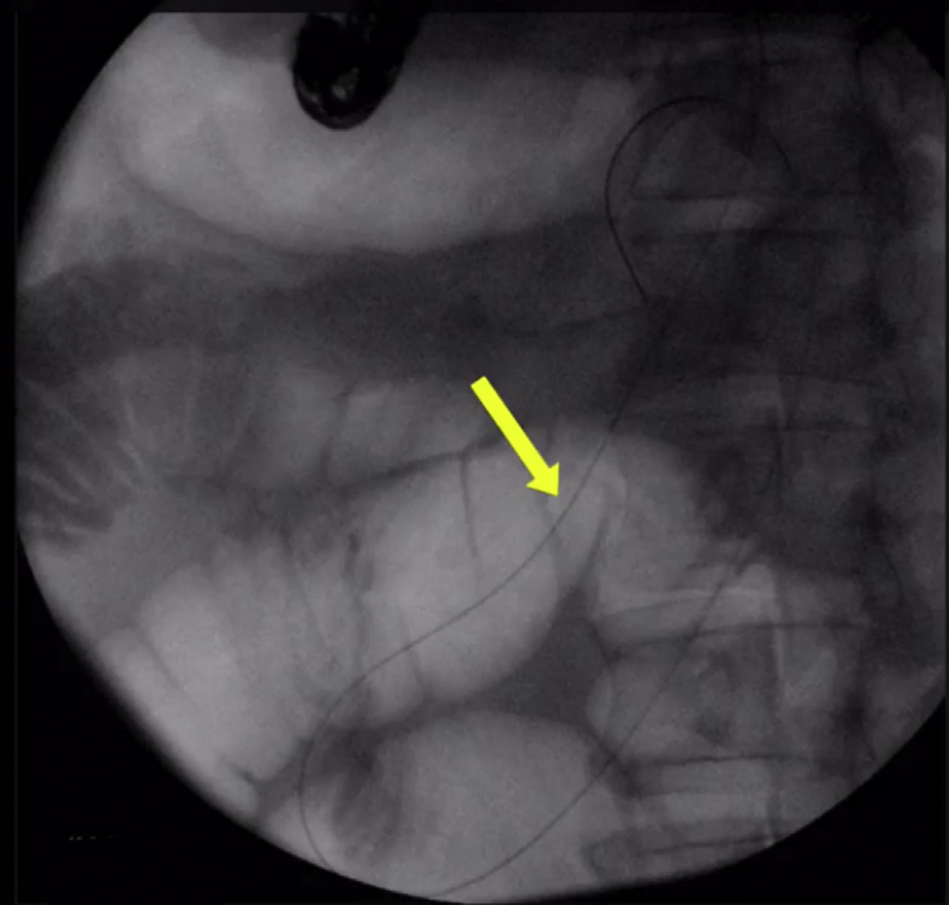
A long wire was manipulated downstream through the ampulla and coiled in the duodenum (*yellow arrow*).

**Figure 5:**
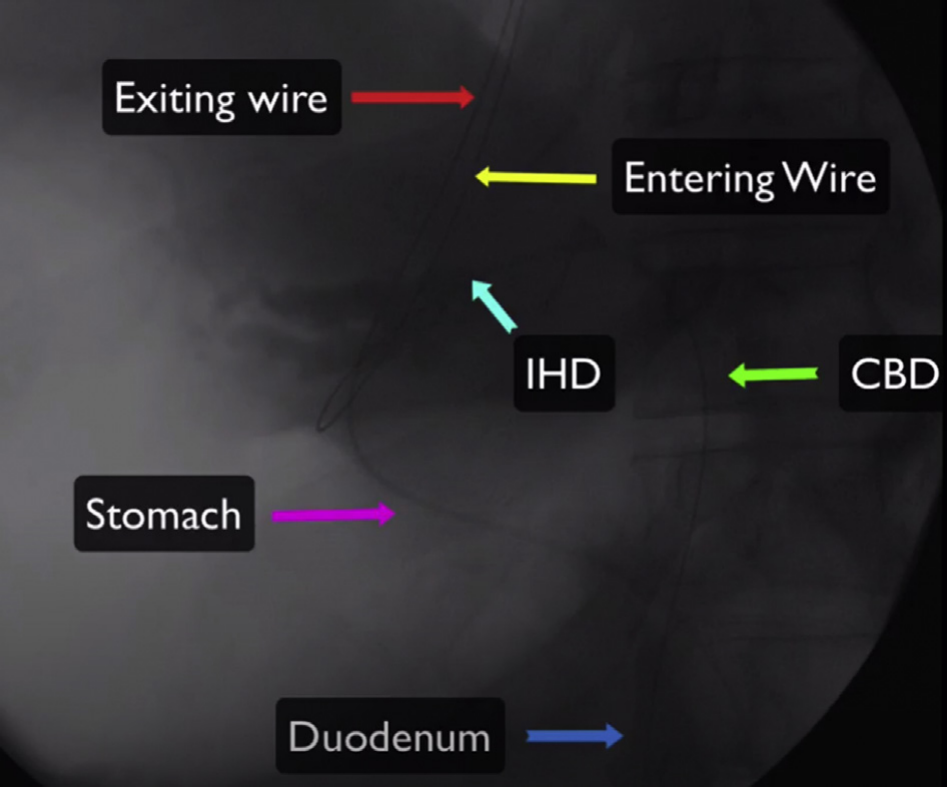
The wire loop after controlling the 2 ends of the wire at the mouth.

**Figure 6:**
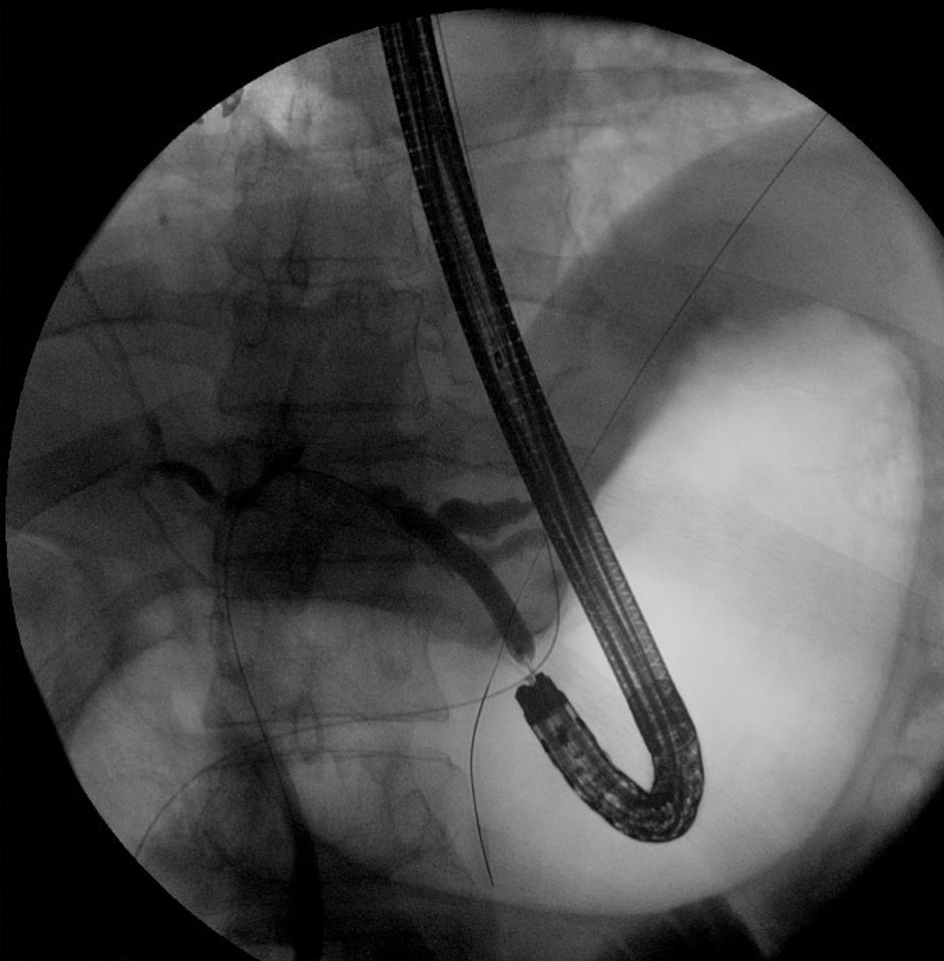
Dilating the hepaticogastrostomy tract using a hurricane balloon.

**Figure 7:**
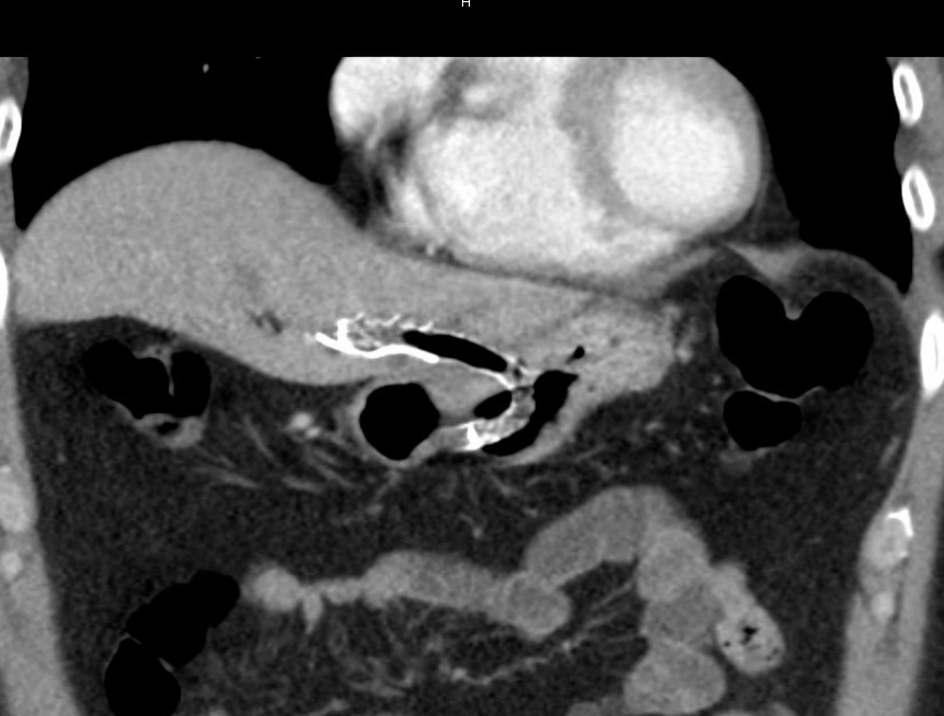
CT image of the Viabil stent across the hepaticogastrostomy.

The wire was manipulated successfully downstream through the papilla (Fig. 4). The echoendoscope was removed, and a duodenoscope was advanced to the papilla; the wire was grasped and pulled out through the mouth without wire entanglement (Fig. 5). A therapeutic endoscope was then advanced over the “entering” end of the wire to the site of the hepaticogastrostomy. We used gentle tension on the “exiting” end of the wire, and the dilating devices were able to penetrate the hepaticogastrostomy; the tract was dilated using a dilating balloon (Fig. 6). After that, a fully covered, 8-mm × 10-cm stent was deployed (Fig. 7).

**Figure 8:**
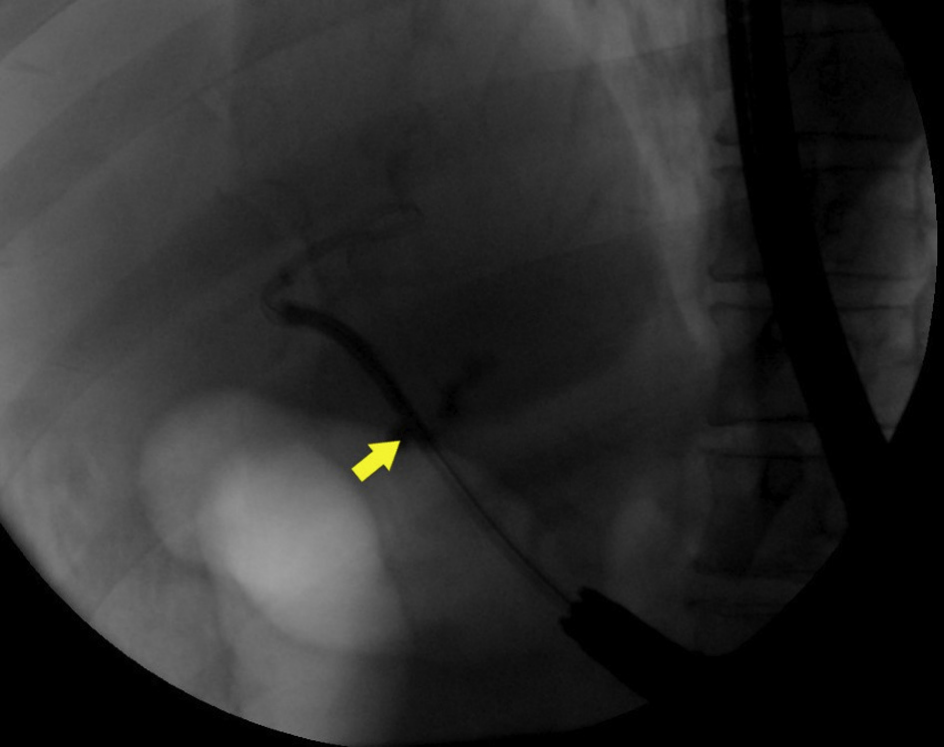
Cholangioscopy of the targeted duct through the hepaticogastrostomy tract.

**Figure 9:**
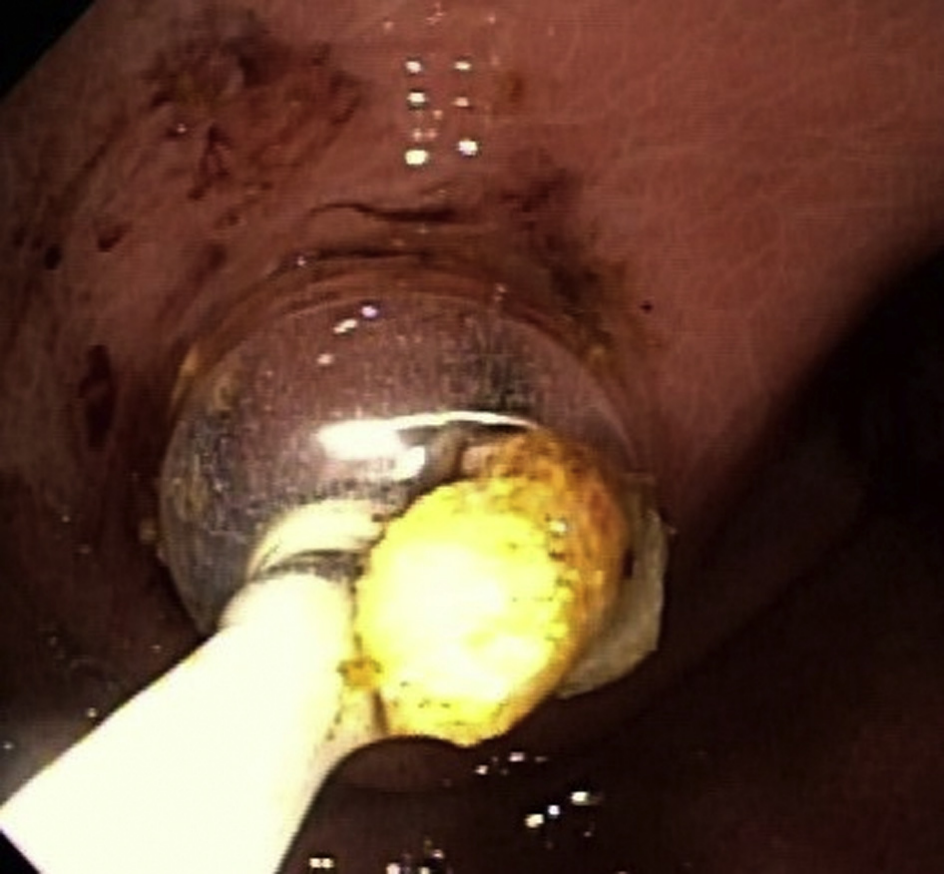
Balloon extraction of stone fragments through the hepaticogastrostomy tract.

Eight weeks later, the patient was brought back for an outpatient procedure. The stent was removed. Cholangioscopy was performed through the hepaticogastrostomy (Fig. 8). Large stones were fragmented using electrohydraulic lithotripsy and were removed (Fig. 9). Another stent was placed.

Two weeks later, cholangioscopy showed a few stones, which were extracted. Two weeks after that, a final cholangioscopy showed no residual stones. No stent was placed at that time, allowing the hepaticogastrostomy to close.

## Outcome

The rendezvous procedure resulted in shredding a small piece of wire that was successfully retrieved during cholangioscopy. Otherwise, no adverse events were noted. Follow-up MRCP 6 months after the index procedure demonstrated decompression of the targeted duct without stones (Fig. 1B). The patient remained asymptomatic (Video 1, available online at www.giejournal.org).

## Disclosure

*All authors disclosed no financial relationships.*

## Supplementary data

Video 1 EUS-guided hepaticogastrostomy using a rendezvous technique to enable cholangioscopy and electrohydraulic lithotripsy of left intrahepatic duct stones.
